# Efficient removal of antibody aggregates using TOYOPEARL MX-Trp-650M mixed-mode resin under salt or pH gradient elution

**DOI:** 10.14440/jbm.0029

**Published:** 2025-07-30

**Authors:** Jinyi Zhang, Penglong Zhang, Yifeng Li

**Affiliations:** Downstream Process Development, WuXi Biologics, Waigaoqiao Free Trade Zone, Shanghai 200131, China

**Keywords:** Aggregate, Antibody, Capto MMC ImpRes, Mixed-mode resin, MX-Trp-650M, pH gradient, Salt gradient

## Abstract

**Background:**

TOYOPEARL MX-Trp-650M is a mixed-mode resin (Tosoh Bioscience, Japan), which mediates both cation exchange and hydrophobic interactions. While mixed-mode resins are generally effective at removing antibody aggregates, reports specifically evaluating the application of MX-Trp-650M for this purpose remain limited. A previous study suggested that effective separation of monomeric and aggregated antibodies using MX-Trp-650M was achieved only under dual-gradient elution with pH and salt.

**Objective:**

This study aimed to further evaluate MX-Trp-650M’s aggregate separation potential under various elution conditions.

**Methods:**

In an antibody purification case where aggregates were the predominant byproducts, both Capto MMC ImpRes and MX-Trp-650M were evaluated as the first polishing step following Protein A capture. Aggregate separation was monitored and assessed using native gel electrophoresis and size-exclusion chromatography-high-performance liquid chromatography.

**Results:**

MX-Trp-650M marginally outperformed Capto MMC ImpRes and achieved excellent aggregate clearance under either salt or pH mono-gradient elution.

**Conclusion:**

Combining the results from prior studies, the current data confirm the strong aggregate separation capability of MX-Trp-650M. The results also suggest that the optimal elution conditions for efficacious separation may vary across different antibody purification scenarios.

## 1. Introduction

TOYOPEARL MX-Trp-650M represents a mixed-mode resin produced by Tosoh Bioscience, which uses tryptophan as the active ligand.^1^,^2^ As tryptophan contains carboxyl and indole groups, MX-Trp-650M mediates both cation exchange and hydrophobic interactions. pH and salt concentrations are the primary factors influencing protein binding and separation on MX-Trp-650M. At a given salt concentration, the binding capacity of MX-Trp-650M greatly depends on pH. Under a moderate conductivity (~12 mS/cm), the highest dynamic binding capacity (DBC) is observed between pH 4.3 and 4.7, reaching approximately 90 mg/mL when tested with human immunoglobulin G.^2^ DBC decreases sharply as pH increases to above 4.7 (e.g., ~30 mg/mL at pH 5.0).^2^ MX-Trp-650M is capable of separating the two monospecific homodimers from the target heterodimer when employed to purify a bispecific κλ-body.^3^ Specifically, under the selected loading conditions, mono λ did not bind, whereas the target κλ-body and mono κ bound with different strengths and were sequentially eluted under 75 mM and 500 mM sodium chloride (NaCl), respectively. In addition, MX-Trp-650M has been utilized to separate intact insulin-like growth factor-1 (IGF-1) from disulfide-scrambled IGF-1 oligomers.^4^ These two studies demonstrated that MX-Trp-650M had excellent selectivity.

As mixed-mode resins combine at least two distinct interaction modes (e.g., electrostatic and hydrophobic interactions), they normally show improved selectivity and aggregate separation capability. For example, both Capto MMC ImpRes and Capto adhere, two mixed-mode resins from Cytiva, have demonstrated strong aggregate separation capabilities.^5^-^11^ MX-Trp-650M shares some similar features with Capto MMC ImpRes, and both resins’ ligands contain carboxyl and aromatic groups. Interestingly, despite their similarity and the wide application of Capto MMC for aggregate removal, reports on the application of MX-Trp-650M for aggregate clearance are scarce. In a study by Tosoh Bioscience,^12^ an mAb sample containing 17% of aggregates was processed using an MX-Trp-650M column. The sample was loaded at pH 4.0 in the presence of 200 mM NaCl. Increasing NaCl concentration to 500 mM failed to elute the protein, and a pH gradient from 4.0 to 12.0 in the presence of 200 mM NaCl resulted in a single elution peak without resolution. Effective separation of aggregates from monomers was only attained when a pH change (from 4.0 to 5.6) was accompanied by a simultaneous NaCl gradient (from 200 to 400 mM). In another report, a pH-salt dual gradient was also applied when MX-Trp-650M was used to remove aggregates.^13^

Recently, our attempt using Capto MMC ImpRes as the first post-Protein A polishing step on an aggregation-prone antibody for aggregate clearance resulted in a suboptimal aggregate reduction. This partial success prompted us to try the MX-Trp-650M. While the previously recommended pH-salt dual gradient elution did not accomplish effective aggregate separation, salt or pH mono gradient elution allowed for good separation of aggregates from monomers. The observations made in the current study, in combination with data from previous studies, not only confirm that MX-Trp-650M is a valuable tool for aggregate clearance but also suggest that conditions for effective separation can vary substantially among different cases.

## 2. Materials and methods

### 2.1. Materials

Ethanol, glycerol, sodium acetate trihydrate, and NaCl were purchased from Merck (Germany). Acetic acid was procured from J.T. Baker (USA). Sodium hydroxide was from Hunan Erkang (China). 30% acrylamide and potassium hydroxide came from Sigma-Aldrich (USA). β-alanine was bought from Accela ChemBio (China). Ammonium persulfate (APS) was purchased from Shanghai Hushi Chemical (China). N,N,N’,N’-tetramethylethylenediamine (TEMED) and methyl green were products from Sangon Biotech (China). eStain LG staining and destaining solutions were purchased from GenScript (China). Capto MMC ImpRes resin and Superdex 200 were made by Cytiva (Sweden). TOYOPEARL MX-Trp-650M resin and TSKgel G3000SWxl stainless steel column (7.8 × 300 mm) were obtained from Tosoh (Japan). The 96-well filter plate with a 0.45 μm polyether sulfone membrane was from Pall (USA). The target antibody (isoelectric point: 7.4) used in this study was expressed in stably transfected CHO-K1 cells grown in home-made culture medium supplemented with Cytiva HyClone Cell Boost 7a and 7b (Sweden). The cells were cultivated for 14 days before harvest.

### 2.2. Equipment

An AKTA pure 150 system installed with Unicorn software version 7.8 (Cytiva, Sweden) was used for Capto MMC ImpRes and MX-Trp-650M column chromatography. A Tecan Freedom EVO 200 (Tecan, Switzerland) was employed for measurement of partition coefficient (K_p_). Measurements of pH and conductivity were performed using a SevenExcellence S470 pH/conductivity meter (Mettler-Toledo, USA). Protein concentration was measured on a NanoDrop 2000 spectrophotometer (Thermo Fisher Scientific, USA). An Agilent 1260 liquid chromatography instrument (Agilent Technologies, USA) was used for size-exclusion chromatography-high-performance liquid chromatography (SEC-HPLC). A bioreactor system from Applikon Biotechnology (the Netherlands) was utilized for cell cultivation.

### 2.3. Capto MMC ImpRes chromatography

Capto MMC ImpRes resin was packed into a column with a diameter of 0.5 cm and a bed height of 14.7 cm. The column volume (CV) was approximately 2.9 mL. As the first polishing step post capture, the column was loaded with Protein A eluate (pH adjusted to 5.5) at 30 mg of protein per mL of resin. After loading, the column was washed with 50 mM sodium acetate-acetic acid (NaAc-HAc), pH 5.5. Subsequently, the column was eluted under a linear salt gradient (A: 50 mM NaAc-HAc, pH 5.5; B: 50 mM NaAc-HAc, 0.5 M NaCl, pH 5.5; 0 – 100% B over 20 CV). The flow rate was set at 176 cm/h (residence time: 5 min).

### 2.4. TOYOPEARL MX-Trp-650M chromatography

MX-Trp-650M was packed into a column with a diameter of 0.5 cm and a bed height of 15.0 cm. The CV was roughly 2.9 mL. To assess MX-Trp-650M’s potential as an alternative to Capto MMC ImpRes for aggregate separation, the column was loaded with the same Protein A eluate at pH 4.5 in the presence of 150 mM NaCl (loading density: 30 mg of protein per mL of resin). After loading, the column was washed with 100 mM NaAc-HAc, 150 mM NaCl, pH 4.5. Initially, the column was eluted under a linear pH-salt dual gradient (A: 100 mM NaAc-HAc, 150 mM NaCl, pH 4.5; B: 100 mM NaAc-HAc, 350 mM NaCl, pH 6.0; 0 – 100% B over 20 CV). After observing that the linear pH-salt dual gradient failed to provide effective aggregate separation, we tried linear salt gradient elution (A: 100 mM NaAc-HAc, 150 mM NaCl, pH 4.5; B: 100 mM NaAc-HAc, 350 mM NaCl, pH 4.5; 0 – 100% B over 20 CV), which provided good aggregate clearance. Next, a stepwise salt gradient elution (100 mM NaAc-HAc, 320 mM NaCl, pH 4.5) was developed based on the linear gradient result. In addition, linear and stepwise pH gradient elutions (A: 100 mM NaAc-HAc, 150 mM NaCl, pH 4.5, B: 100 mM NaAc-HAc, 150 mM NaCl, pH 5.3; 0 – 100% B over 20 CV and 100 mM NaAc-HAc, 150 mM NaCl, pH 5.1, respectively) were also developed. For all runs, the system was run at a flow rate of 180 cm/h (residence time: 5 min).

### 2.5. Preparative size-exclusion chromatography

To obtain monomers and aggregates for the K_p_ measurement, preparative SEC was performed using a Superdex 200 column (inner diameter: 5.0 cm, bed height: 87.7 cm, CV: ~1722 mL). The column was equilibrated with 50 mM Tris-HAc, 150 mM NaCl, pH 7.4, for 1.5 CV and then loaded with materials pre-purified by Protein A affinity chromatography to a volume of about 5.8% of the CV. After loading, the column was washed with the equilibration buffer for 1.5 CV. For all steps, the column was run at a flow rate of 15 cm/h.

### 2.6. Partition coefficient measurement for monomer and aggregates

Partition coefficient, defined as the ratio of resin-bound protein to free protein in solution at equilibrium, was used to evaluate the binding strength of monomer and aggregates to MX-Trp-650M and Capto MMC ImpRes under various conditions. Experiments were conducted on an automated liquid handling system (Tecan Freedom EVO 200) with a 96-well filter plate, where each well was loaded with 20 μL of resin and equilibrated with buffers at predetermined pHs (4.5 and 5.5 for MX-Trp-650M and Capto MMC ImpRes, respectively) and NaCl concentrations (i.e., 200, 250, 300, 350, 400 mM for MX-Trp-650M, and 200, 300, 400, 500, 600 mM for Capto MMC ImpRes). SEC prepared monomers, adjusted to the corresponding pH and NaCl concentration (to a final protein concentration of 1.0 mg/mL), were added at 5 mg protein per mL of resin (100 μL per well), followed by agitation at 1,300 rpm for 30 min. After incubation, the supernatant was analyzed in terms of ultraviolet (UV) absorbance at 280 nm to determine the concentration of unbound protein. For each selected condition, K_p_ measurement was conducted in duplicate.

### 2.7. SEC-HPLC

SEC-HPLC was performed using a TSKgel G3000SWxl column (7.8 × 300 mm) from Tosoh Bioscience, Japan. The mobile phase was 50 mM sodium phosphate buffer (pH 6.8) containing 300 mM NaCl. 100 μg of sample was injected per run, and elution was conducted isocratically at a flow rate of 1.0 mL/min. Protein was monitored in terms of UV absorbance at 280 nm.

### 2.8. Acidic native gel electrophoresis

Acidic native (non-denaturing) gel electrophoresis, serving as a complementary tool to SEC-HPLC for monitoring antibody aggregates, was performed using home-cast gels consisting of stacking and resolving portions. The stacking gel (3.0%) was prepared by mixing 0.2 mL of 30% acrylamide, 0.5 mL of 4X stacking gel buffer (240 mM KOH, 238 mM HAc, pH 6.8), 1.28 mL of purified water, 20 μL of 10% APS, and 2 μL of TEMED. The resolving gel (10.0%) was made by combining 1.67 mL of 30% acrylamide, 1.25 mL of 4X resolving gel buffer (240 mM KOH, 726 mM HAc, pH 4.3), 1.15 mL of 50% glycerol, 0.88 mL of purified water, 50 μL of 10% APS, and 5 μL of TEMED. For sample analysis, 2 μg of protein was mixed with 5X loading buffer (36% w/w glycerol, 26% w/w 4X stacking buffer, and 0.5% w/w methyl green) and purified water to dilute the loading buffer to a final concentration of 1X. Electrophoresis was conducted at 180 V for 3 h by using a running buffer containing 350 mM β-alanine and 133 mM acetic acid (pH 4.3). Since the target antibody is positively charged under acidic conditions and migrates toward the cathode, it is critical to reverse the polarity of the leads to avoid sample loss. Upon completion of the electrophoresis, the gel was stained and destained with eStain LG solutions (GenScript) according to the manufacturer’s instructions.

## 3. Results and discussion

### 3.1. Notable but suboptimal aggregate clearance using Capto MMC ImpRes

**Figure 1:**
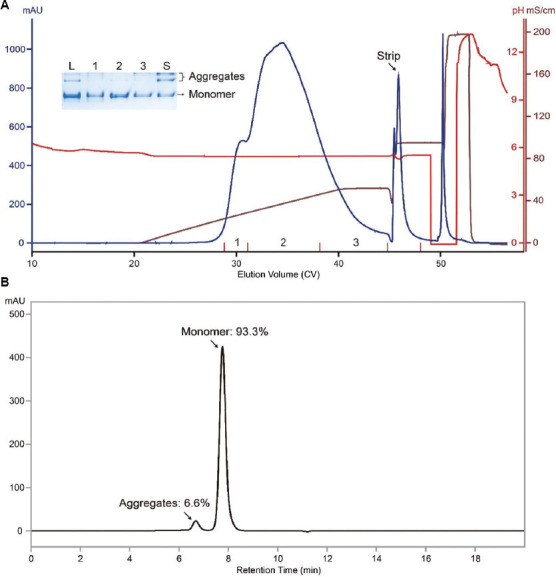
Capto MMC ImpRes run data obtained under linear salt gradient elution. (A) Capto MMC ImpRes chromatogram of a run conducted under linear salt gradient elution. The load material was Protein A eluate, which contained 15.3% of aggregates. (B) SEC-HPLC profile of a sample aliquot from the Capto MMC ImpRes elution pool. The percentages of monomer and aggregates are labeled. Notes: Inset: Native gel analysis of relevant fractions. L: Load material; Lanes 1 – 3: Elution fractions 1 – 3; S: Strip. Bands corresponding to monomeric and aggregated antibodies are indicated. The main elution fraction contained two bands, which likely represent glycosylation isoforms, the same reason that led to peak splitting. Abbreviation: SEC-HPLC: Size-exclusion chromatography-high-performance liquid chromatography.

Capto MMC ImpRes is a mixed-mode resin from Cytiva, and its ligand contains both cation exchange and hydrophobic moieties.^14^-^16^ In several previous studies, we demonstrated that Capto MMC ImpRes is a powerful tool for aggregate clearance.^5^-^7^ Recently, we found that the aggregates in Protein A eluate accounted for 15.3% of the total mass during the purification of an aggregation-prone antibody, as indicated by SEC-HPLC analysis. Therefore, the aggregates need to be reduced to an acceptable level by polishing steps. Based on previous successful experience with Capto MMC ImpRes in removing aggregates, this resin was initially selected for post-Protein A polishing. The bound antibody was eluted under linear salt gradient elution, and the chromatogram is shown in Figure 1A. The elution profile contains split peaks. Further analysis suggests that peak splitting is due to the existence of glycosylation isoforms (data not shown). Reduction of aggregates in the eluate was indicated by native gel analysis of relevant fractions (Figure 1A). SEC-HPLC analysis of a sample aliquot from the entire elution pool indicated that aggregate content was lowered to 6.6% (Figure 1B). Although there was a notable reduction of aggregates, the results were still suboptimal, as previous studies utilizing Capto MMC ImpRes managed to reduce aggregate content to a much lower level, with a similar level of aggregates in the feed.^5^-^7^

### 3.2. Limited resolution between monomers and aggregates under linear pH-salt dual gradient elution using MX-Trp-650M

**Figure 2:**
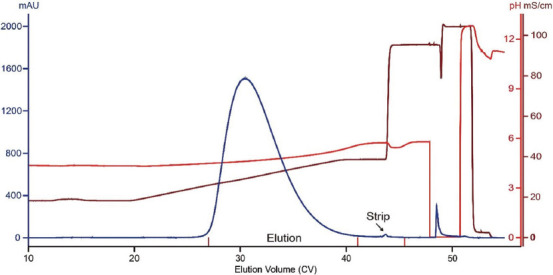
MX-Trp-650M chromatogram of a run conducted under linear pH-salt dual gradient elution. The load material was Protein A eluate, which contained 15.3% of aggregates.

We next evaluated MX-Trp-650M as an alternative to Capto MMC ImpRes for post-Protein A polishing using the same feed material (i.e., Protein A eluate containing 15.3% of aggregates). As prior studies suggested that effective separation of monomers from aggregates by MX-Trp-650M was only achieved under pH-salt dual gradient elution, we first tested an elution condition comparable to that used in the previous report (i.e., a pH increase from 4.5 to 6.0 accompanied by a salt concentration increase from 150 to 350 mM over 20 CV). The chromatogram of this run is shown in Figure 2. The profile contains a single elution peak and a tiny strip peak, suggesting that the separation of monomers from aggregates is unlikely. Poor aggregate separation was confirmed by SEC-HPLC analysis of the elution pool sample, which indicated that the elution pool contained 14.6% of aggregates (profile not shown).

### 3.3. Effective separation of monomers from aggregates under salt or pH mono gradient elution using MX-Trp-650M

**Figure 3:**
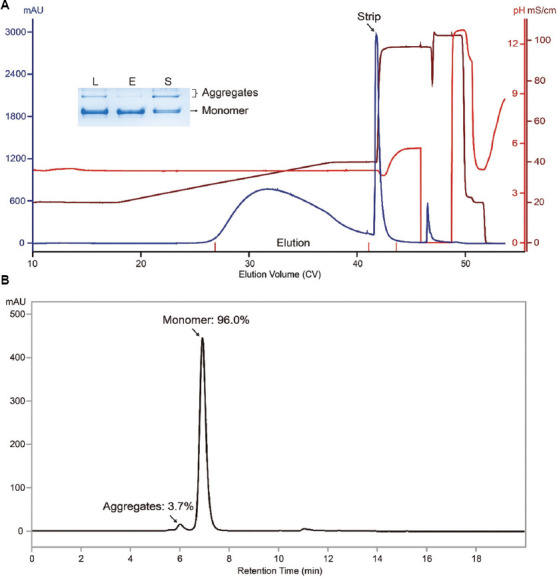
MX-Trp-650M run data obtained under linear salt gradient elution. (A) MX-Trp-650M chromatogram of a run conducted under linear salt gradient elution. The load material was the same as that used for the run conducted under pH-salt dual gradient elution. (B) SEC-HPLC profile of a sample aliquot from the MX-Trp-650M elution pool. The percentages of monomer and aggregates are labeled. Note: Inset: Native gel analysis of relevant fractions. L: Load material; E: Elution pool; S: Strip. Bands corresponding to monomeric and aggregated antibodies are indicated. Abbreviation: SEC-HPLC: Size-exclusion chromatography-high-performance liquid chromatography.

In the dual gradient elution, changes in pH and salt concentration both promoted protein elution. It was suspected that this condition was too strong to obtain a good resolution between monomers and aggregates. Therefore, we tested linear salt mono gradient elution (from 150 to 350 mM) under a defined pH (i.e., 4.5). The chromatogram of this run is shown in Figure 3A. The profile is remarkably different from that obtained under the pH-salt dual gradient elution. In particular, the profile contains a large strip peak, indicating the separation of aggregates. Effective aggregate removal under this condition was confirmed by native gel and SEC-HPLC of relevant fractions and a sample aliquot of the elution pool, respectively (Figures 3A and B). The SEC-HPLC data indicated that aggregate content in the elution pool was reduced to 3.7%. The step yield of this run was 74.7% (monomer recovery: ~85.0%). In comparison to Capto MMC ImpRes, MX-Trp-650M not only provided better aggregate clearance but also avoided peak splitting.

**Figure 4:**
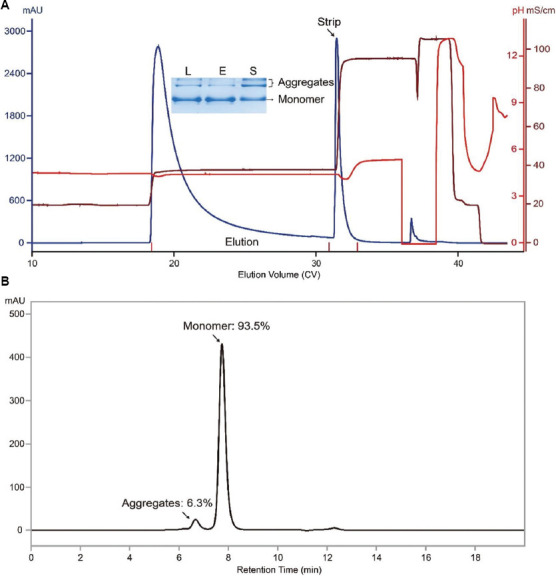
MX-Trp-650M run data obtained under stepwise salt gradient elution. (A) MX-Trp-650M chromatogram of a run conducted under stepwise salt gradient elution. The load material was the same as that used for the run conducted under linear salt gradient elution. (B) SEC-HPLC profile of a sample aliquot from the MX-Trp-650M elution pool. The percentages of monomer and aggregates are labeled. Note: Inset: Native gel analysis of relevant fractions. L: Load material; E: Elution pool; S: Strip. Bands corresponding to monomeric and aggregated antibodies are indicated. Abbreviation: SEC-HPLC: Size-exclusion chromatography-high-performance liquid chromatography.

As the data suggested, under linear salt gradient elution, aggregates were well separated from monomers and were mainly found in the strip. This facilitated the development of stepwise salt gradient elution (320 mM NaCl, pH 4.5). The chromatogram of the run conducted under the stepwise salt gradient elution is shown in Figure 4A. Both native gel and SEC-HPLC analysis of the MX-Trp-650M eluate indicated that a good aggregate clearance was maintained (Figures 4A and B). Specifically, aggregate content was reduced to 6.3%. In addition, under the stepwise salt gradient elution, the MX-Trp-650M step yield was 74.2% (monomer recovery: ~82.2%). Thus, under stepwise gradient elution, both purity and yield are inferior to those obtained under linear gradient elution. This is not surprising as linear gradient elution generally provides a better resolution than its stepwise counterpart.

**Figure 5:**
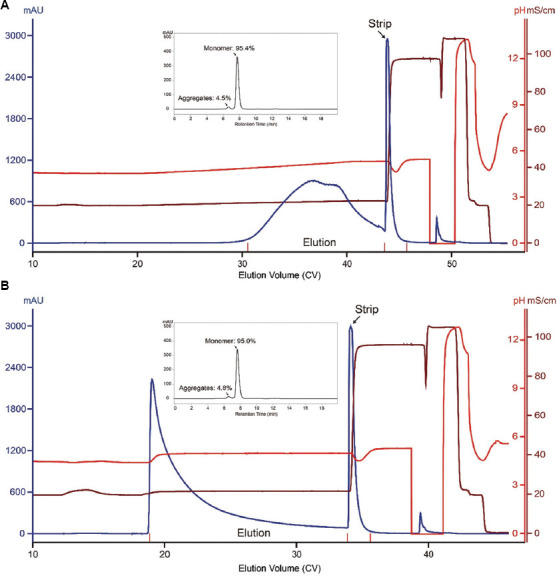
MX-Trp-650M chromatograms of runs conducted under linear pH and stepwise pH gradient elution. (A) Linear pH. (B) Stepwise pH gradient elution. The load material was the same as that used for runs conducted under salt gradient elution. Note: Inset: SEC-HPLC profile of a sample aliquot from the corresponding MX-Trp-650M elution pool. The percentages of monomer and aggregates are labeled. Abbreviation: SEC-HPLC: Size-exclusion chromatography-high-performance liquid chromatography.

Encouraged by the promising separation achieved by MX-Trp-650M under salt gradient elution, we further tested pH gradient elution under a defined salt concentration (i.e., 150 mM NaCl). The chromatograms obtained under linear and stepwise pH gradient (pH 4.5 – 5.3 and pH 5.1, respectively) elution are shown in Figures 5A and B, respectively. According to the SEC-HPLC data of the corresponding elution pool, aggregate content was lowered to 4.5% and 4.8% under these two conditions, respectively (Figures 5A and B). The step yields under these two conditions were 75.1% and 69.5%, and the corresponding monomer recoveries were ~85.0% and ~78.2%, respectively. Overall, pH gradient elution provides results comparable to those obtained under salt gradient elution (the lower percentage of aggregates seen under stepwise pH gradient was obtained at the sacrifice of yield).

### 3.4. Further comparison between MX-Trp-650M and Capto MMC ImpRes

While the ligands of MX-Trp-650M and Capto MMC ImpRes both carry carboxylic acid and a hydrophobic moiety, the aromatic groups in their hydrophobic moieties are indole and benzyl, respectively. Therefore, they are different in their hydrophobic nature. In addition, while MX-Trp-650M achieves high DBC within a narrow pH range (i.e., 4.3 – 4.7), Capto MMC ImpRes shows good DBC over a wide pH range (i.e., 5.0 – 8.0).^2^,^14^-^16^ In this case, the measured DBC for MX-Trp-650M and Capto MMC ImpRes under their favourable pHs (4.5 and 5.5, respectively) are 71 mg/mL and 69 mg/mL, respectively. The aforementioned difference between these two resins may contribute to their unique performances under linear salt gradient elution observed in the current study, which are similar but distinct.

**Figure 6:**
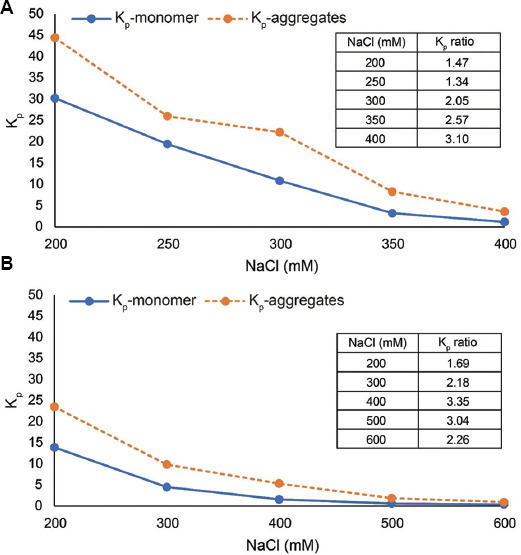
The plot of K_p_-monomer and K_p_-aggregates under various salt concentrations (conductivities) using two different mixed-mode resins. (A) MX-Trp-650M, pH 4.5. (B) Capto MMC ImpRes, pH 5.5. For each resin, K_p_ ratios under each salt concentration are given in the table within the corresponding figure.

For mixed-mode media that mediate cation exchange and hydrophobic interactions, conductivity gradient elution is more frequently utilized than pH gradient elution. In addition, the separation potential of a given medium under a specific condition can be estimated in terms of the ratio of K_p_ of the two species that are intended to be separated.^17^-^19^ Thus, to further compare the potential of MX-Trp-650M and Capto MMC ImpRes for aggregate separation, we separately measured the K_p_ for monomer and aggregates at different salt concentrations using each resin under their favorable pHs (4.5 and 5.5, respectively). The plots are shown in Figure 6. For both resins under each salt concentration tested, the separation factor, defined as the ratio between K_p_-aggregates and K_p_-monomer,^17^-^19^ was calculated. The values are listed in the table within the corresponding plot (Figure 6). A larger ratio is indicative of better separation potential. For both resins, the largest separation factor was obtained under 400 mM NaCl (3.10 and 3.35 for MX-Trp-650M and Capto MMC ImpRes, respectively). The K_p_ ratios suggest that these two mixed-mode resins possess similar aggregate separation potential. However, the K_p_ data indicate that both the monomer and aggregates exhibit stronger binding to MX-Trp-650M compared to Capto MMC ImpRes, a difference that may slightly enhance the separation of these two species by the former resin.

## 4. Conclusion

Aggregate removal is a common challenge facing the downstream processing of recombinant antibodies. In general, aggregate removal relies on post-capture polishing steps, and mixed-mode resins are more powerful than traditional single-mode resins in this regard.^5^-^11^ In the current study, we demonstrated that the MX-Trp-650M column could significantly reduce aggregates when used as the first polishing step post-Protein A capture (remaining aggregates could be further reduced to an acceptable level by the second polishing step). Interestingly, effective aggregate removal was achieved under salt or pH mono gradient elution rather than pH-salt dual-gradient elution, which was recommended by all previous studies. Additionally, in the current study, MX-Trp-650M performed better than Capto MMC ImpRes, a similar mixed-mode resin known for its strong aggregate separation capability. Data from the current and previous studies suggest that, for a given mixed-mode resin, the condition for effective aggregate separation can vary remarkably across different molecules. For a given molecule, the performance of similar mixed-mode resins can vary. As multiple distinct but interacting interactions are involved in binding mediated by a mixed-mode resin, it is not surprising that a small change/difference can result in appreciably different outcomes. Thus, to find the best mixed-mode resin for separation and harness the full potential of each mixed-mode resin, it is highly recommended to systematically screen different resins under various conditions, and the screening can be performed using high-throughput approaches.^9^,^17^,^18^,^20^,^21^

## Data Availability

Data are available upon request from the corresponding author.

## References

[1] Tosoh Bioscience. TOYOPEARL MX-Trp-650M. Available from: https://www.separations.eu.tosohbioscience.com/OpenPDF.aspx?path=~/File%20Library/TBG/Products%20Download/Flyer/f22p01a.pdf. [Last accessed on 2025 Jun 09].

[2] Tosoh Bioscience. Development of a High Capacity Mixed-Mode Resin for High Conductivity Mab Feedstocks. Application Note. Available from: https://www.bioprocessintl.com/monoclonal-antibodies/development-of-a-high-capacity-mixed-mode-resin-for-high-conductivity-mab-feedstocks. [Last accessed on 2025 Jun 09].

[3] Fouque N Depoisier JF Wilson K Vajda J Muller E Dabre R. Methods of Purifying Bispecific Antibodies. US 10,457,749 B2. United States Patent. 2019.

[4] Arakawa T. Isoform separation by a mixed-mode resin, TOYOPEARL MX-Trp-650M. Curr Protein Pept Sci. 2019;20 1:61-64. 10.2174/1389203718666171009111355. 28990530

[5] Tang J Zhang X Chen T Wang Y Li Y. Removal of half antibody, hole-hole homodimer and aggregates during bispecific antibody purification using MMC ImpRes mixed-mode chromatography. Protein Expr Purif. 2020;167:105529. 10.1016/j.pep.2019.105529. 31698035

[6] Wan Y Zhang T Wang Y Wang Y Li Y. Removing light chain-missing byproducts and aggregates by Capto MMC ImpRes mixed-mode chromatography during the purification of two WuXiBody-based bispecific antibodies. Protein Expr Purif. 2020;175:105712. 10.1016/j.pep.2020.105712. 32738441

[7] Chen T Guo G Tan G Wang Y Li Y. Antibody aggregate removal using a mixed-mode chromatography resin. Methods Mol Biol. 2021;2178:345-354. 10.1007/978-1-0716-0775-6_23. 33128760

[8] Zhang L Parasnavis S Li Z Chen J Cramer S. Mechanistic modeling based process development for monoclonal antibody monomer-aggregate separations in multimodal cation exchange chromatography. J Chromatogr A. 2019;1602:317-325. 10.1016/j.chroma.2019.05.056. 31248584

[9] Altern SH Lyall JY Welsh JP. High-throughput in silico workflow for optimization and characterization of multimodal chromatographic processes. Biotechnol Prog. 2024;40 6:e3483. 10.1002/btpr.3483. 38856182

[10] Gao D Wang LL Lin DQ Yao SJ. Evaluating antibody monomer separation from associated aggregates using mixed-mode chromatography. J Chromatogr A. 2013;1294:70-75. 10.1016/j.chroma.2013.04.018. 23639130

[11] Robinson J Vats M Hartmann M. Implementation of multimodal anion exchange chromatography to address product quality challenges and downstream platform limitations:A case study. J Chromatogr A. 2025;1746:465784. 10.1016/j.chroma.2025.465784. 39983562

[12] Vajda J. Separation of monoclonal immunoglobulin G and its aggregates using TOYOPEARL MX-Trp-650M. Bioprocess Int. 2013;2013 -2014:73-75.

[13] Vajda J Mueller E Bahret E. Dual salt mixtures in mixed mode chromatography with an immobilized tryptophan ligand influence the removal of aggregated monoclonal antibodies. Biotechnol J. 2014;9 4:555-565. 10.1002/biot.201300230. 24421277

[14] Cytiva. Polishing of Monoclonal Antibodies Using Capto MMC ImpRes in Bind and Elute Mode, Application Note, 2013, 29-0373-49 AA. Available from: https://cdn.cytivalifesciences.com/api/public/content/digi-16467-pdf. [Last accessed on 2025 Jun 09].

[15] Cytiva. Capto MMC ImpRes, Datafile, 2015, 29-035674 AB. Available from: https://gels.yilimart.com/assets/images/doc/file/17371601_datafile_01.pdf. [Last accessed on 2025 Jun 09].

[16] Cytiva. Capto MMC ImpRes Resin. Available from: https://cdn.cytivalifesciences.com/api/public/content/digi-16468-pdf. [Last accessed on 2025 Jun 09].

[17] Kramarczyk JF Kelley BD Coffman JL. High-throughput screening of chromatographic separations:II. Hydrophobic interaction. Biotechnol Bioeng. 2008;100 4:707-720. 10.1002/bit.21907. 18496875

[18] Wensel DL Kelley BD Coffman JL. High-throughput screening of chromatographic separations:III. Monoclonal antibodies on ceramic hydroxyapatite. Biotechnol Bioeng. 2008;100 5:839-854. 10.1002/bit.21906. 18551522

[19] Kelley BD Tobler SA Brown P. Weak partitioning chromatography for anion exchange purification of monoclonal antibodies. Biotechnol Bioeng. 2008;101 3:553-566. 10.1002/bit.21923. 18727127

[20] McDonald P Tran B Williams CR. The rapid identification of elution conditions for therapeutic antibodies from cation-exchange chromatography resins using high-throughput screening. J Chromatogr A. 2016;1433:66-74. 10.1016/j.chroma.2015.12.071. 26803905

[21] Ram J Snyder M Belisle C Koley S Vecchiarello N. Defining operating regimes for partition coefficient measurements in protein chromatography. J Chromatogr A. 2025;1745:465730. 10.1016/j.chroma.2025.465730. 39919683

